# Evolving Toward Non-narcotic Perioperative Enhanced Recovery After Surgery and Opioid-Free Analgesia in the Management of Postoperative Pain

**DOI:** 10.7759/cureus.76605

**Published:** 2024-12-30

**Authors:** Justin H Wong, Yujin Na, Fereydoun D Parsa

**Affiliations:** 1 Department of Medicine, University of Hawaii John A. Burns School of Medicine, Honolulu, USA; 2 Department of Surgery, Division of Plastic Surgery, University of Hawaii John A. Burns School of Medicine, Honolulu, USA

**Keywords:** beta-endorphin, enhanced recovery after surgery, opioid-free analgesics, opioid substitution, postoperative pain management

## Abstract

Opioid medications are commonly employed for perioperative and postoperative pain management. However, these medications can negatively impact the body's innate pain management system, specifically the action of beta-endorphins. By impairing the function of mu-opioid receptors and inhibiting the release of beta-endorphin, opioids may exacerbate and prolong postoperative pain. Additionally, opioid use is associated with numerous side effects, including nausea, vomiting, constipation, excessive sedation, clouded sensorium, dizziness, respiratory depression, and addiction, all of which may impede postoperative patient recovery and outcome quality. The purpose of this article is to explore the intricate relationship between opioid medications and endogenous beta-endorphins, examine nonopioid modalities for postoperative pain control, and elucidate the applications of non-narcotic perioperative enhanced recovery after surgery protocols in improving patient outcomes.

## Introduction and background

The National Institute on Drug Abuse (NIDA) reports that more than 280,000 people died in the United States from 1999 to 2021 due to an overdose involving prescription opioids [1]. Deaths related to prescription opioids were five times higher in 2021 compared to 1999 [1]. In the past decade, fentanyl use and prescription have increased in US health systems, contributing to an increase in overdose mortality, with adolescents experiencing a greater relative increase compared to the overall population [2,3]. Although opioid use and expenditures in health systems have decreased in the past decade, the number of deaths related to opioids remains high [3,4].

Opioid analgesics such as hydrocodone, oxycodone, and fentanyl are routinely used in perioperative and postoperative pain control, which may increase the risk of opioid abuse [4-6]. Opioid analgesics are associated with significant adverse effects including nausea, vomiting, constipation, excessive sedation, insomnia, clouded sensorium, dizziness, respiratory depression, and addiction [5,7]. Numerous studies have previously shown that minimizing opioid use perioperatively aids in controlling acute postoperative pain, reduces opioid consumption, improves patient length of stay, decreases the risk of postoperative nausea/vomiting, and leads to fewer unplanned hospital admissions [5-20]. Using multimodal opioid-free analgesics (OFA) as the next step in pain management may reduce opioid prescriptions, improve patient recovery, and reduce overall healthcare resource utilization and costs [21].

The harmful effects of opioids and their associated rise in overdose mortality rates highlight the need for nonopioid modalities for pain control. Enhanced recovery after surgery (ERAS) protocols have been demonstrated to be equally or more effective than opioid-based regimens in managing postoperative pain, resulting in shorter hospital stays and improved patient outcomes [4-26]. These protocols employ an evidence-based, holistic approach to treatment, minimizing the use of opioid analgesics to enhance patient recovery and satisfaction. However, despite their proven effectiveness, many physicians continue to rely heavily on opioid analgesics [17,18]. This article aims to review the interplay between opioid medications and endogenous beta-endorphins, discuss nonopioid modalities for postoperative pain control, and illustrate the applications of non-narcotic perioperative ERAS protocols on patient outcomes.

## Review

Opioid analgesics and beta-endorphins

Exogenous opioid analgesics are commonly employed in both perioperative and postoperative pain management. These opioids exert their analgesic effects by modulating signal transduction pathways both presynaptically and postsynaptically [9,27]. Opioid receptors, which are G protein-coupled receptors, play a pivotal role in this process. Activation of these receptors leads to the blockade of presynaptic calcium channels and the opening of postsynaptic potassium channels, ultimately inhibiting the release of neurotransmitters such as substance P and glutamate, thereby reducing the transmission of pain signals [9,27-29].

Of the various opioid receptors, mu-opioid receptors are the primary targets for prescription opioids like hydrocodone, oxycodone, morphine, and fentanyl [27,28]. These medications mimic the actions of endogenous endorphins, particularly beta-endorphins, by binding to mu-opioid receptors present in both the central nervous system (CNS) and peripheral nervous system [5,27-29]. However, the use of exogenous opioids can have detrimental effects on the body's natural pain response mechanisms. It inhibits the production of beta-endorphins and downregulates mu-opioid receptors through mechanisms such as decreased proopiomelanocortin gene expression and receptor uncoupling [5,28]. Beta-endorphins are estimated to be 18 to 33 times more potent analgesics than morphine [5,7].

Chronic opioid use poses additional risks, including opioid-induced hyperalgesia (OIH), tolerance, and addiction, owing to alterations in neural plasticity [7,28]. Consequently, the prolonged and intensified postoperative pain may result from impairment in the body's innate pain response mechanism due to exogenous opioid use. Recognizing these risks underscores the importance of transitioning towards OFA as a progressive approach in pain management.

Opioid-free analgesics

Opioid complications can lead to increased addiction, mortality rates, prolonged hospital stays, higher risks of readmission, and elevated healthcare costs among surgical patients [5,9,18,21,22,25]. Given the ongoing opioid crisis and its detrimental consequences, there is an urgent need to explore OFA for effective postoperative pain management. Selected OFA that may be used instead of opioids are now briefly summarized as follows: acetaminophen, nonsteroidal anti-inflammatory drugs (NSAIDs), gabapentinoids, ketamine, intravenous lidocaine, and paravertebral nerve blocks.

Acetaminophen

Acetaminophen exerts its analgesic effects by inhibiting the cyclooxygenase pathway in the CNS [5,18,22]. Metabolized primarily in the liver, a single dose can offer up to 50% pain relief for four hours in patients with moderate-to-severe acute postoperative pain [22]. Intravenous administration of acetaminophen boasts more favorable pharmacokinetics and bioavailability compared to oral and rectal routes due to minimal first-pass hepatic metabolism [18,22]. Generally well-tolerated with minimal adverse effects, acetaminophen is widely utilized for postoperative pain management [5,18,20,22] and has been linked to decreased opioid requirements during the postoperative period [5,20].

Nonsteroidal anti-inflammatory drugs

Nonsteroidal anti-inflammatory drugs (NSAIDs) function by inhibiting the enzymatic activity of cyclooxygenase-1 (COX-1) and/or cyclooxygenase-2 (COX-2), thus disrupting prostaglandin synthesis and yielding analgesic, antipyretic, and anti-inflammatory effects [3,19,20]. These drugs have been shown to decrease opioid consumption and may synergize with acetaminophen to enhance pain management [5,22]. While nonselective NSAIDs like ibuprofen and ketorolac are generally well-tolerated, they can lead to adverse effects such as gastrointestinal (GI) ulceration, bleeding, and renal impairment [22]. On the other hand, selective COX-2 inhibitors like celecoxib offer GI protection and may lower the risk of GI bleeding; however, they are associated with increased risks of myocardial infarction, stroke, and renal failure [5,20,22]. NSAIDs have proven efficacy in pain management, but their potential for side effects warrants cautious use.

Gabapentinoids

Gabapentinoids, such as gabapentin and pregabalin, are antiepileptic medications that exert their analgesic effects by inhibiting voltage-gated calcium channels and modulating arachidonic acid, nitrergic, and serotonergic systems [5,18,22]. The perioperative administration of gabapentin has been associated with reductions in acute postoperative pain, opioid consumption, nausea, and vomiting [13,22]. These benefits must be weighed against potential adverse effects, including sedation, visual disturbances, and lightheadedness, which may impede early postoperative mobilization and delay recovery [3,20]. Despite their promise, the current understanding of the perioperative use of gabapentinoids remains limited, highlighting the need for further research to establish clear dosing guidelines [9,18,22].

Ketamine

Ketamine, a dissociative anesthetic, acts by inhibiting N-methyl-D-aspartate (NMDA) receptors in both the brain and spinal cord, thereby reducing the transmission of pain signals and inducing a potent anesthetic and analgesic response [9,22,24,30]. When administered intravenously, ketamine has demonstrated the ability to reverse opioid tolerance, leading to reduced opioid consumption and improved pain control [22,30]. Numerous studies have consistently reported positive outcomes associated with perioperative ketamine administration, including decreased incidence of postoperative nausea and vomiting, as well as enhanced pain management and overall patient recovery [10,14,24]. While ketamine offers significant therapeutic benefits, it is important to note its common adverse effects, such as nausea, vomiting, dizziness, diplopia, drowsiness, dysphoria, and confusion [22,30]. Nevertheless, ketamine's potential to prevent opioid tolerance and the development of OIH suggests that it may hold particular promise for patients who are opioid tolerant [22].

Intravenous lidocaine

Lidocaine, an amide local anesthetic, exerts its analgesic and anti-inflammatory effects by inhibiting voltage-gated sodium channels [18,22]. Previous studies have demonstrated that lidocaine infusion can effectively reduce postoperative pain, opioid consumption, nausea, vomiting, and duration of hospital stays, while also promoting improved patient recovery [14,22]. Despite these promising findings, the lack of comprehensive knowledge regarding optimal dosing and infusion duration poses challenges in fully confirming the benefits of perioperative and postoperative lidocaine use in pain management [22]. Therefore, further research is warranted to address these gaps and provide clearer insights into the potential advantages of lidocaine in pain control.

Paravertebral nerve blocks

Paravertebral nerve blocks (PNBs) offer analgesic benefits by directly targeting peripheral nerves with local anesthetics [22,25]. Continuous PNBs, providing a sustained duration of analgesia compared to single-shot PNBs, have been associated with enhanced postoperative outcomes [22]. Previous research has shown that PNBs contribute to decreased postoperative opioid consumption, as well as reduced incidences of nausea and vomiting, and improved patient recovery and satisfaction [5-6,9,22,25]. However, it is important to note that PNBs, acting across sensory, motor, and sympathetic domains within multiple dermatomes, may delay postoperative ambulation, potentially leading to prolonged hospital stays [6,25]. Complications associated with PNBs include hypotension, bleeding, nerve injury, infection, pneumothorax, and epidural spread [5-6,22]. Nevertheless, the utilization of ultrasound guidance for PNB placement has been shown to mitigate these risks and enhance block precision [6,22].

Enhanced recovery after surgery protocols

ERAS protocols created by the ERAS Society in 1997 have been widely adopted across various surgical subspecialties, initially starting with colorectal surgery and later expanding to include vascular, hepatobiliary, thoracic, urologic, gynecologic, and plastic surgery [4,8,26]. In light of the ongoing opioid crisis and the associated risks of drug abuse, it is crucial to explore safer alternatives for postoperative pain management [2,4,8,10,11,22,23,25,26]. While the literature emphasizes the avoidance of opioid administration, ERAS encompasses a comprehensive approach that includes preoperative counseling, multimodal non-narcotic analgesia, and early postoperative ambulation [5,18,22,23]. In addressing the escalating healthcare costs and the prevalence of opioid abuse, ERAS offers a safer, more cost-effective solution for both providers and patients, while also proving to be more efficacious in managing postoperative pain [2,18,21,23].

Preoperative counseling

Rather than merely focusing on the absence of opioids, effective implementation of ERAS protocols necessitates surgeons to reassess their approach to counseling patients regarding postoperative pain expectations and management plans [18,21]. By accounting for individual variations in pain tolerance, preoperative counseling has been shown to significantly improve patient satisfaction and reduce postoperative opioid consumption [5,20]. While opioids effectively alleviate somatic pain, their impact on neuropathic and inflammatory pain, which are less understood, remains negligible [5,18]. Educating patients about the mechanism of opioids and their paradoxical inhibitory effect on endogenous beta-endorphins, the body's natural mechanism for pain control and mood enhancement, has resulted in significantly reduced opioid use and more favorable postoperative reports of mood and discomfort levels [5,18,20,26,28]. Essentially, patients who received preoperative explanations about the expected pain and discomfort following surgery reported experiencing less pain and improved mood.

Multimodal analgesia

The ongoing opioid crisis underscores the urgent need for multimodal analgesia (MMA) [4,11,22,31]. MMA involves the use of analgesic medications from multiple classes, targeting different pain receptors. This approach has demonstrated efficacy in improving postoperative pain management while minimizing adverse effects associated with the singular use of opioids [31]. Despite the widespread use of opioids, studies indicate that 80% of patients receiving opioids report inadequate treatment of postoperative pain [11,22]. Ineffective analgesia, along with the harmful after-effects and increased overdose mortality associated with opioids, underscores the necessity for advancements in nonopioid modalities for postoperative pain management. MMA is particularly promising due to its potential for individualized treatment according to each patient's unique pain profile.

A comprehensive MMA protocol may include a combination of opioids, OFA such as NSAIDs, gabapentinoids, ketamine, and local anesthetics [9,18,31,32]. Among OFA medications, acetaminophen is widely used due to its evidence-based history of effective pain management and opioid-sparing mechanism [5,9,18,31]. Co-administration of acetaminophen and NSAIDs has been recommended to maximize analgesic efficacy, particularly in the initial 48 hours post-surgery [18,32]. Additionally, NSAIDs combined with local anesthetics, such as bupivacaine, have been shown to significantly reduce inflammation, a major contributor to postoperative pain [18]. The synergistic effect of gabapentin and celecoxib in pain reduction is also supported. Although further research is needed, the administration of dexamethasone with ondansetron has shown promise in reducing overall inflammation and providing effective antiemetic therapy [12].

Early postoperative ambulation

Early postoperative ambulation, often overlooked but crucial within ERAS protocols, refers to a patient's ability to walk independently or with minimal assistance, a pivotal indicator of postoperative recovery [33]. Surgeons have long advocated for early postoperative ambulation, recognizing its role in stimulating digestive processes and alleviating gastroparesis [4,33,34]. Studies assessing time to ambulation, patient recovery, mobility, morbidity rates, and hospital length of stay have consistently demonstrated that patients undergoing ERAS protocols achieve faster ambulation, earlier hospital discharge, and enhanced recovery outcomes [13,18,33,34]. Postoperative mobilization typically includes activities like transitioning from bed to chair and walking in the room or hallway, with ERAS protocols delineating specific objectives for distances walked or time spent out of bed [35]. As a safe, cost-effective element of ERAS, it is imperative not to overlook the importance of early postoperative ambulation in postoperative pain management.

Clinical considerations

Amidst the ongoing opioid crisis, the escalating rates of deaths from prescription opioid overdose over the past decade underscore the urgent need for new strategies to curb physician opioid prescription practices and mitigate the incidence of opioid abuse [36]. The widespread use of exogenous opioids for perioperative and postoperative pain management, although common, is not without its drawbacks. While these medications may offer temporary relief, their ability to inhibit the production of beta-endorphins and downregulate mu-opioid receptors can exacerbate and prolong postoperative pain. Furthermore, the array of adverse effects associated with opioid consumption can significantly impede overall patient recovery and outcomes.

In line with the 2022 Centers for Disease Control and Prevention (CDC) guidelines for opioid prescribing, the emphasis is placed on prioritizing nonopioid therapies whenever feasible [36]. When opioid prescription is unavoidable, physicians are urged to exercise caution, prescribing the lowest effective dose to mitigate the risks of opioid use disorder and overdose. Fortunately, there exists a multitude of opioid-free alternatives for pain management, with potential benefits in enhancing patient recovery while minimizing adverse effects. Parsa et al. (2020) reported extensive experience in utilizing OFA perioperatively and postoperatively for patients undergoing plastic surgery procedures, successfully avoiding opioid use in nearly all cases over the past two decades [7,20].

Due to the more personalized nature of ERAS in postoperative pain management, more time, attention, and care are required of surgeons to implement ERAS protocols in a befitting manner. ERAS was introduced less than 30 years ago, and as such, the traditional methods taken for treating postoperative pain have proven difficult to veer away from; 70% of ERAS patients reporting minimal pain in immediate hospitalization were given a prescription for opioids upon discharge [3,9,23]. Other research found the incidence of opioid prescriptions at discharge to be lower in patients with ERAS protocol implementation; however, the resultant drop in opioid prescriptions did not hold statistical significance when compared to the pre-ERAS implementation patients [3,4]. Albeit gradual, implementation of ERAS protocols consistently exhibits decreased reports of postoperative pain, decreased nausea, fatigue, and drowsiness on postoperative days 0-1, as well as quicker postoperative ambulation [4,9,23]. Results such as these, all in the absence of opioids, demonstrate the weight ERAS protocols carry in enhancing postoperative care holistically [23]. 

While this article provides expert opinion and synthesizes current knowledge through a literature review of the use of ERAS protocols and OFA in pain management, it is important to acknowledge its limitations. The lack of case-control analysis in this article limits its ability to provide empirical evidence for the effectiveness of these interventions.

## Conclusions

The widespread adoption of ERAS protocols and OFA across various surgical specialties and postoperative pain management settings underscores the feasibility of reducing or eliminating opioid use in routine clinical practice. Nevertheless, physicians must remain vigilant in assessing individual patient needs and judiciously considering when opioid use may be warranted while maintaining cautious dosing practices. Clinicians can continue to advance toward the goal of minimizing opioid reliance in pain management by prioritizing effective patient communication and education on the adverse effects of exogenous opioid use and OFA options. The efficacy and long-term benefits of nonopioid modalities in improving postoperative patient outcomes warrant further investigation.
